# tsRNA-04002 alleviates intervertebral disk degeneration by targeting PRKCA to inhibit apoptosis of nucleus pulposus cells

**DOI:** 10.1186/s13018-023-03878-3

**Published:** 2023-06-07

**Authors:** Jie Pan, Zhonghan Liu, Bin Shen, Jin Xu, Gonghua Dai, Wen Xu, Jianjie Wang, Lijun Li, Liming Cheng

**Affiliations:** 1 Department of Spinal Surgery, Shanghai East Hospital, School of Medicine, Tongji University, Shanghai, 200092 China; 2 Department of Anesthesiology, Shanghai East Hospital, School of Medicine, Tongji University, Shanghai, 200092 China; 3 Department of Medical Imaging, Shanghai East Hospital, School of Medicine, Tongji University, Shanghai, 200092 China; 4 Department of Orthopedics, Tongji Hospital, School of Medicine, Tongji University, No.389 Xincun Road, Putuo Distrcit, Shanghai, 200092 China

**Keywords:** Intervertebral disk degeneration, Apoptosis, Inflammatory cytokines, Nucleus pulposus cells, tsRNA-04002, PRKCA

## Abstract

**Background:**

Intervertebral disk degeneration (IDD) is a degenerative disease that underlies various musculoskeletal and spinal disorders and is positively correlated with age. tRNA-derived small RNAs (tsRNA), as a new small noncoding RNAs, its function in IDD is unclear. Herein, our goal was to find the key tsRNA that affects IDD independently of age and explore the underlying mechanisms.

**Methods:**

Small RNA sequencing was performed in nucleus pulposus (NP) tissues of traumatic lumbar fracture individuals, young IDD (IDDY) patients, and old IDD (IDDO) patients. The biological functions of tsRNA-04002 in NP cells (NPCs) were investigated by qRT-PCR, western blot, and flow cytometry analysis. The molecular mechanism of tsRNA-04002 was demonstrated by luciferase assays and rescue experiments. Furthermore, the therapeutic effects of tsRNA-04002 on IDD rat model were used and evaluated in vivo.

**Results:**

Compared with fresh traumatic lumbar fracture patients, a total of 695 disordered tsRNAs is obtained (398 down-regulated tsRNAs and 297 up-regulated tsRNAs). These disordered tsRNAs were mainly involved in Wnt signaling pathway and MAPK signaling pathway. tsRNA-04002 was an age-independent key target in IDD, which was both lower expressed in IDDY and IDDO groups than control group. Overexpression of tsRNA-04002 restrained inflammatory cytokines IL-1*β* and TNF-*α* expression, increased the COL2A1, and inhibited the NPCs apoptosis. Furthermore, we determined that PRKCA was the target gene of tsRNA-04002 and was negatively regulated by tsRNA-04002. The rescue experiment results suggested that the high expression of PRKCA reversed the inhibitory effect of tsRNA-04002 mimics on NPCs inflammation and apoptosis, and promotive effect of COL2A1. Moreover, tsRNA-04002 treatment dramatically ameliorated the IDD process in the puncture-induced rat model, together with the blockade of PRKCA in vivo.

**Conclusion:**

Collectively, our results substantiated that tsRNA-04002 could alleviate IDD by targeting PRKCA to inhibit apoptosis of NPCs. tsRNA-04002 may be a novel therapeutic target of IDD progression.

**Supplementary Information:**

The online version contains supplementary material available at 10.1186/s13018-023-03878-3.

## Introduction

Intervertebral disk degeneration (IDD) is the basis of a variety of musculoskeletal and spinal diseases, such as structural instability, intervertebral disk (IVD) protrusion, spinal stenosis [1]. IDD usually induces lower back pain (LBP) [2]. LBP is the skeletal muscle system disease with the highest incidence rate in the world [3]. More than 85% of adults maybe suffer from LBP, and LBP is one of the main causes of disability [4]. However, the pathogenesis of IDD is still unclear. Consequently, it is necessary to explore the pathogenesis of IDD for the treatment of IDD.

The IVD is composed of the central gelatinous nucleus pulposus (NP) and the surrounding annulus fibrosus (AF), while NP and AF are sandwiched by the cartilage endplate [5]. NP cells (NPCs) are located in NP, responsible for the synthesis and maintenance of gel-like extracellular matrix (ECM), such as collagen type II (COL2A1), which are the main functional components of IVD in the face of various external mechanical compression [6]. IDD features include accelerated ECM degradation, produced proinflammatory mediators, reduced height of IVD, and increased cell loss, aging, and death [5, 7]. Inflammation also plays an important role in IDD. Increased levels of inflammatory cytokines such as interleukin-1*β* (IL-1*β*) and tumor necrosis factor-*α* (TNF-*α*) were observed in degenerative IVD tissues [8]. Increased expression of IL-1*β* and TNF-*α* induced ECM degradation, increased the production of the catabolic factor matrix metalloproteinase (MMP), inhibited the expression of the anabolic factors COL2A1, and induced excessive apoptosis of NPCs leading to IVD degeneration [9, 10]. However, the efficacy of various clinical treatments is very limited. Moreover, the current treatment methods are limited to relieving IDD symptoms, not aiming at the pathophysiology of IDD [11]. New therapies such as tissue engineering and cell therapy are still at the stage of laboratory exploration [12]. Therefore, exploring the changes in the molecular pathogenesis of IDD provides new ideas for IDD treatment and diagnosis.

tRNA-derived small RNAs (tsRNA) are a new type of regulatory small noncoding RNAs, which participate in a variety of physiological and pathological processes [13]. In the animal model of ischemia–reperfusion, the production of tsRNA was proportional to the degree of ischemia–reperfusion injury and cell death; tsRNA had good potential to be a biomarker of stroke [14]. Moreover, tsRNA could be a novel epigenetic molecule regulating adipogenesis [15]. Meanwhile, sperm tsRNAs help to transmit acquired metabolic disorders to the next generation [16]. Moreover, tsRNA has received wide attention in recent years in the fields of reproduction, nerve and tumor. tsRNA was a candidate humoral biomarker for gender-dependent Parkinson's disease [17]. However, there is no report on the relationship between tsRNA and IDD. The miRNA with similar structures to tsRNA, which have been common in IDD research [18–20]. Based on this, we speculated that tsRNA is important for IDD development. PRKCA (protein kinase C Alpha) is a protein-coding gene. Cui et al. reported that PRKCA could induce apoptosis [21]. In addition, PRKCA has been shown to have a significant inhibitory anti-inflammatory effect on some diseases, such as acute lung injury [22]. Although apoptosis and inflammatory responses of NPCs are critical in the pathogenesis of IDD, the role of PRKCA in IDD remains unknown.

Here, we determined the tsRNA expression profile by small RNA sequencing in NP tissue samples from young IDD (IDDY) patients and old IDD (IDDO) patients and controls. Next, we explored the function of an age-independent key target on IDD and clarified its molecular mechanism in regulating NPCs apoptosis, which may offer a new insight into the diagnosis and therapy of IDD.

## Materials and methods

### Tissue samples collection

The NP tissue samples were collected from IDDY patients during discectomy (IDDY group, mean age 25 years), IDDO patients during discectomy (IDDO group, mean age 67 years), and fresh traumatic lumbar fracture patients (normal group, mean age 22 years) (*n* = 3). All patients underwent magnetic resonance imaging and were graded according to Pfirrmann classification. Normal tissue was rated as grade I and IDDY/IDDO tissue as grade III–V. The experiments of human were approved by the Medicine Ethics Committee of Shanghai East Hospital Affiliated to Tongji University (2022-037), and the written informed consent of patients was obtained.

### Small RNA sequencing

Total RNA was extracted from the collected clinical NP tissues to construct a small RNA library. In brief, 3 and 5 adapters were added to both ends of the RNA, followed by PCR amplification. The amplified products were purified using QIAquick PCR Purification Kit and then subjected to Agilent 2100 Bioanalyzer (Agilent Technologies, USA) to determine the RNA. IlluminaHiseq Hiseq2500 platform was used for sequencing. The sequencing raw data were filtered, mapping and quantitative processing.

### Bioinformation analysis

The obtained tsRNA was annotated, the fragment size and distribution were analyzed, the differential tsRNA expression profile was analyzed according to the threshold (Log2FC > 1 or <  − 1, *p* value < 0.05). The differential tsRNA target genes were predicted by Miranda and RNAhybrid. The GO function enrichment and KEGG pathway analysis were performed for functional enrichment analysis of differentially expressed tsRNAs. Cytoscape software 3.6.1 was applied to figure interaction network of the tsRNA- mRNA-pathway.

### NPCs isolation and culture

NPCs were extracted from fresh patient intervertebral disk tissue. NPCs were cultured in human degenerative nucleus pulposus cell complete medium (CM-H170, Proxil) containing with 5% FBS, 100 mg/mL streptomycin and 100 U/mL penicillin. The cells were passaged when they reached 90% confluence.

### Quantitative reverse transcription PCR (qRT-PCR)

Total RNA was extracted from NP tissues and NPCs using the TRIzol method following the manufacturer’s instructions. Using Reverse Transcription System Kit (Promega), RNA was reverse-transcribed to generate cDNA. The primers were synthesized by TaKaRa, and the PCR amplification was conducted by SYBR Premix Ex Taq. Using U6 or GAPDH as the internal reference gene, the relative expression of the gene to be tested was calculated by the relative quantitative 2^®^^−ΔΔCT^ method. The primers are listed in Additional file 1: Table S1.

### Western blot analysis

NPCs lysates were prepared in RIPA buffer (Thermo) and then extracted protein. Bicinchoninic acid protein assay (Beyotime) was applied for detecting concentrations. Protein was subjected to SDS-PAGE and transferred to a PVDF membrane for immunoblotting analysis. The membranes were incubated with anti-PRKCA (CST, 2056, 1:1000), anti-COL2A1 (Proteintech, 28,459–1-AP, 1:1000) and anti-GAPDH (Proteintech, 60,004–1-Lg, 1:20,000) overnight at 4 °C, and then incubated with goat anti-rabbit IgG-HRP (abcam, ab6721, 1/20000) and goat anti-mouse IgG-HRP (abcam, ab205719, 1:1000) at 24 °C for 2 h. Finally, membranes were exposed to a chemiluminescence imaging analysis and quantified with Image J software.

### Flow cytometry analysis

The Annexin V/PI apoptosis kit (Invitrogen, USA) was applied to detect apoptosis using flow cytometry. The cell suspension was prepared with Annexin V binding solution. Next, cell suspension (1 × 10^6^ cells/mL) was transferred into a culture tube and stained with 1 × Annexin V-FITC and PI. Five μL Annexin V-FITC and 5 μL PI were added into culture tube to incubate with 100 μL cell suspension. The cells were gently vortexed and incubated at room temperature without light for 15 min. Finally, 400 μL 1 × Annexin V binding solution was added and cell apoptosis was detected by flow cytometry.

### Dual luciferase reporter gene assay

HEK293T cells (derived from NANJING COBIOER BIOSCIENCES CO., LTD.) were seeded in 24-well plate before transfection. Then, cells were co-transfected with the tsRNAs (mimic NC, tsRNA-04002 mimic) and luciferase reporter vectors containing tsRNA-04002-PRKCA binding sequences (PRKCA-WT) or mutant sequences (PRKCA-MUT). Then, dual-luciferase reporter assay system (Promega, USA) was analyzed the luciferase activity. The luciferase activity = firefly luciferase activity / renilla luciferase activity.

### Establishment of IDD models

Twenty three-month-old male SD rats (200–250 g, *n* = 5) were randomly divided into three groups: sham group, IDD group and IDD + tsRNA treatment group. The IDD rat model was established by referring to previous studies [23, 24]: the animals were anesthetized with 2% (w/v) pentobarbital (40 mg/kg). After abdominal anesthesia, the rats were prone, the skin was cut with a sharp knife, and the muscle tissue was separated to expose the spinal column and rat tail intervertebral disk (Co7/8). Next, a 21-G (20 mL syringe) needle was used to pierce the tail intervertebral disk at a 45° angle with the spinal column, rotate 180° and stay for 10 s. The skin was sutured, and the wound was covered with gauze. After surgery, IDD rat was subcutaneously injected with 5 nmol tsRNA-04002 agomir or NC agomir at the operation site. To determine whether the modeling was successful, magnetic resonance imaging (MRI) was performed 2 weeks later. All animal protocols were approved by the Animal Care and Use Committee of East Hospital Affiliated to Tongji University.

### Hematoxylin–eosin (HE) staining

After 4 weeks of modeling, the rats were stained with HE. The disk tissues (Co7/8) were collected and then infiltrated in 4% paraformaldehyde at 4 °C for 2 days. Next, decalcification was performed in 10% EDTA. When the bone tissue became soft, decalcification was stopped, followed by gradient ethanol dehydration, paraffin embedding and slicing. Finally, sections were stained with hematoxylin and eosin and observed under light microscopy.

### Safranin O-fast green staining

Safranin O-fast green staining was used after 4 weeks of puncture modeling. The intervertebral disks of rats were fixed in 10% neutral-buffered formalin for 1 week, decalcified with EDTA for 2 weeks, paraffin-embedded, and sectioned. The eosin and safranin O-fast green were performed to stain sections. The tissue morphology was observed by light microscope.

### Immunohistochemical staining

The antigen extracted from paraffin NP tissue sections was added to EDTA buffer for 20 min. Tissue sections were blocked with 5% BSA for 20 min and incubated with anti-PRKCA (1:200) overnight at 4 °C, followed by incubation with goat anti-rabbit IgG for 20 min. The sections were counterstained with hematoxylin and photographed under a microscope.

### Statistical analysis

All data were expressed as mean ± standard deviation. SPSS 21.0 statistical software (IBM Corp. Armonk, NY, USA) was used to analyze data. Independent-sample two-tailed unpaired t-test and one-way analysis of variance (ANOVA) were used for data comparison of two groups and multiple groups, respectively. Drawing column chart with GraphPad 8.0 software, *p* < 0.05 was considered statistically significant.

## Results

### Summary of the tsRNA expression profiles in IDD

The RNA-seq data were first mapped to the human reference genome obtained from the tRFdb database (http://genome.bioch.virginia.edu/trfdb/). As shown in Table 1, after removing the low-quality reads in the raw reads, the IDDY groups obtained 8,470,383, 2,785,897, and 2,193,531 clean reads, respectively; the IDDO groups obtained 2,827,130, 2,863,039, and 3,596,097 clean reads, respectively; and the normal groups gained 4,725,005, 3,071,503, and 3,759,342 clean reads, respectively. These reads mapped to the human genome with an average of 575,989 (12.85% mapping), 1,115,495 (36.04% mapping) and 1,016,206 (26.38% mapping).

**Table 1:** Statistics of the sequencing reads mapping to the reference genome

Samples	Clean reads	Mapped reads	Mapped rate (%)	Clean bases	Mapped bases	Mapped rate (%)
IDDY1	8,470,383	80,684	1.0	210,407,060	2,155,783	1.0
IDDY2	2,785,897	1,066,112	38.3	73,579,745	29,685,319	40.3
IDDY3	2,193,531	581,171	26.5	56,041,321	15,552,456	27.8
IDDO1	2,827,130	851,377	30.1	74,011,369	23,239,495	31.4
IDDO2	2,863,039	1,009,537	35.3	73,079,404	26,078,887	35.7
IDDO3	3,596,097	1,485,570	41.3	95,726,381	40,400,727	42.2
Normal1	4,725,005	262,341	5.6	120,380,491	6,303,700	5.2
Normal2	3,071,503	1,166,753	38.0	80,339,414	32,538,550	40.5
Normal3	3,759,342	1,619,525	43.1	99,160,635	44,712,555	45.1

### Differential expression and functional analysis of tsRNAs

**Fig. 1:**
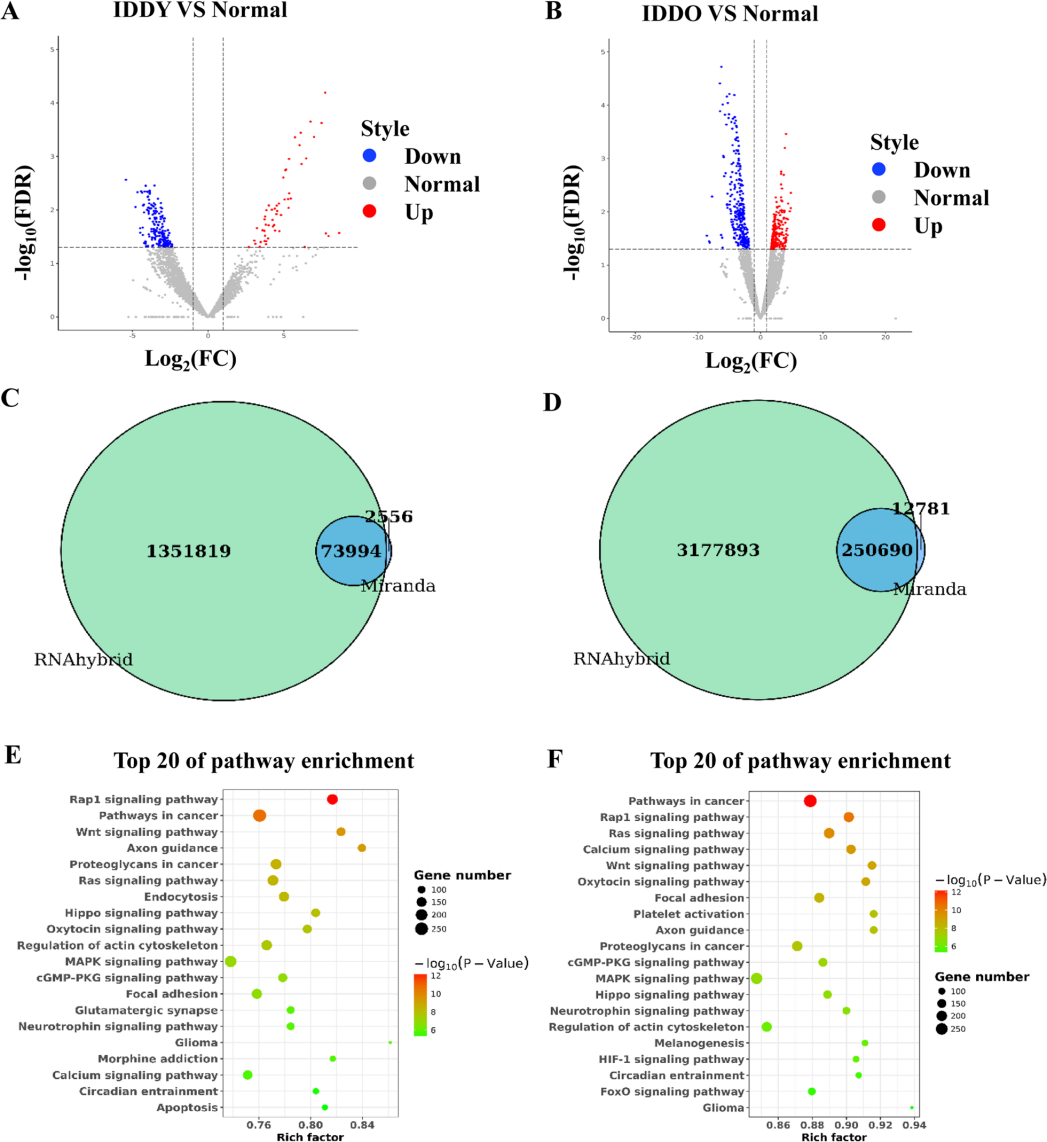
Differential expression and functional analysis of tsRNAs. **A** The volcano plot of tsRNAs in IDDY versus normal. **B** The volcano plot of tsRNAs in IDDO versus normal. **C** RNAhybrid and Miranda predicted target genes of differentially expressed tsRNAs in IDDY versus normal. **D** The target genes of differentially expressed tsRNAs were predicted by RNAhybrid and Miranda in IDDO versus Normal. KEGG pathway analysis for target genes of tsRNAs in IDDY versus normal (**E**) and IDDO versus normal (**F**)

To determine the potential important tsRNAs involved in IDD, we identified the differently expressed tsRNAs between the normal, IDDO and IDDY groups. There were 239 differentially expressed tsRNAs in IDDY versus normal, including 53 up-regulated tsRNAs and 186 down-regulated tsRNAs (Fig. 1A). A total of 546 differentially expressed tsRNAs were identified in the IDDO versus normal, including 245 up-regulated tsRNAs and 301 down-regulated tsRNAs (Fig. 1B). Subsequently, we predicted the target genes of differentially expressed tsRNAs in IDDY versus normal and IDDO versus normal groups and identified 73,994 and 250,690 target genes, respectively (Fig. 1C, D). Simultaneously, KEGG showed that differentially expressed tsRNAs in IDDY versus normal were mainly involved in Rap1 signaling pathway, Wnt signaling pathway and MAPK signaling pathway (Fig. 1E). GO analysis demonstrated that these tsRNAs in IDDY versus normal were enriched in regulation of DNA transcription, RNA translation and protein modification (Additional file 2: Fig. S1A). In addition, we also found that the pathways enriched for target genes of differentially expressed tsRNA between IDDO versus normal were almost exactly similar to the pathways enriched in IDDY versus normal (Fig. 1E and F). Congruously, the biological functions enriched for the target genes of differentially expressed tsRNA between IDDO versus normal were similar to those between DDY versus normal (Additional file 2: Fig. S1A and B). Therefore, we hypothesized that the disorder of tsRNA expression profile was not affected by age. Together, these results showed that the tsRNA expression profile of IDD patients was disordered, which is involved in the development of IDD by regulating the Rap1 signaling pathway, Wnt signaling pathway and MAPK signaling pathway.

### tsRNA-04002 expression is down-regulated in NP tissues of IDD

**Fig. 2:**
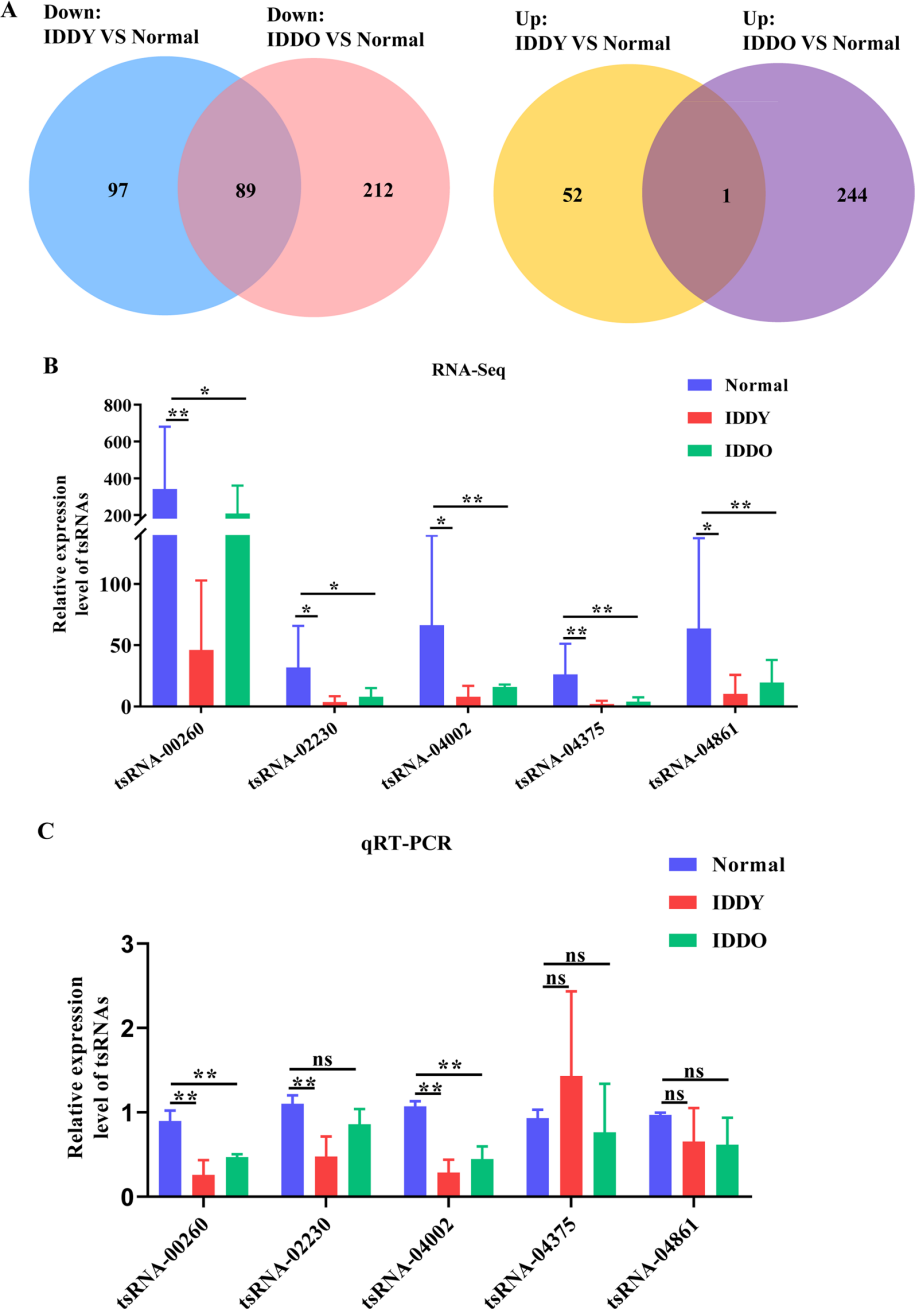
Validation of candidate tsRNAs expression in NP tissues of IDD. **A** Venn diagram showed shared differentially expressed tsRNAs between IDDY versus normal and IDDO versus normal. **B** Five tsRNAs with large fold change and high abundance in RNA-seq. **C** Five tsRNAs were performed for qRT-PCR. *p value < 0.05, **p value < 0.01, *n* = 3

To screen out age-independent key targets in IDD, Venn diagram analysis was performed on the differentially expressed tsRNAs of the three groups. A total of 89 down-regulated genes were obtained by taking the intersection of all down-regulated genes in IDDY versus normal and IDDO versus normal. Similarly, we also obtained 1 up-regulated gene by taking the intersection of all up-regulated genes in IDDY versus normal and IDDO versus normal (Fig. 2A). Subsequently, we selected five tsRNAs with large fold change and high abundance in RNA-seq (Fig. 2B) for qRT-PCR verification. Compared with normal tissues, tsRNA-00260, tsRNA-02230 and tsRNA-04002 were down-regulated in NP tissues of IDDY, and tsRNA-00260 and tsRNA-02230 were down-regulated in NP tissues of IDDO (Fig. 2C). Considering that tsRNA-04002 has the largest fold change compared with the other four tsRNAs and the qRT-PCR results are consistent with the RNA-seq, it is finally determined that tsRNA-04002 is the age-independent tsRNA regulating IDD.

### tsRNA-04002 inhibits inflammation and apoptosis of NPCs

**Fig. 3:**
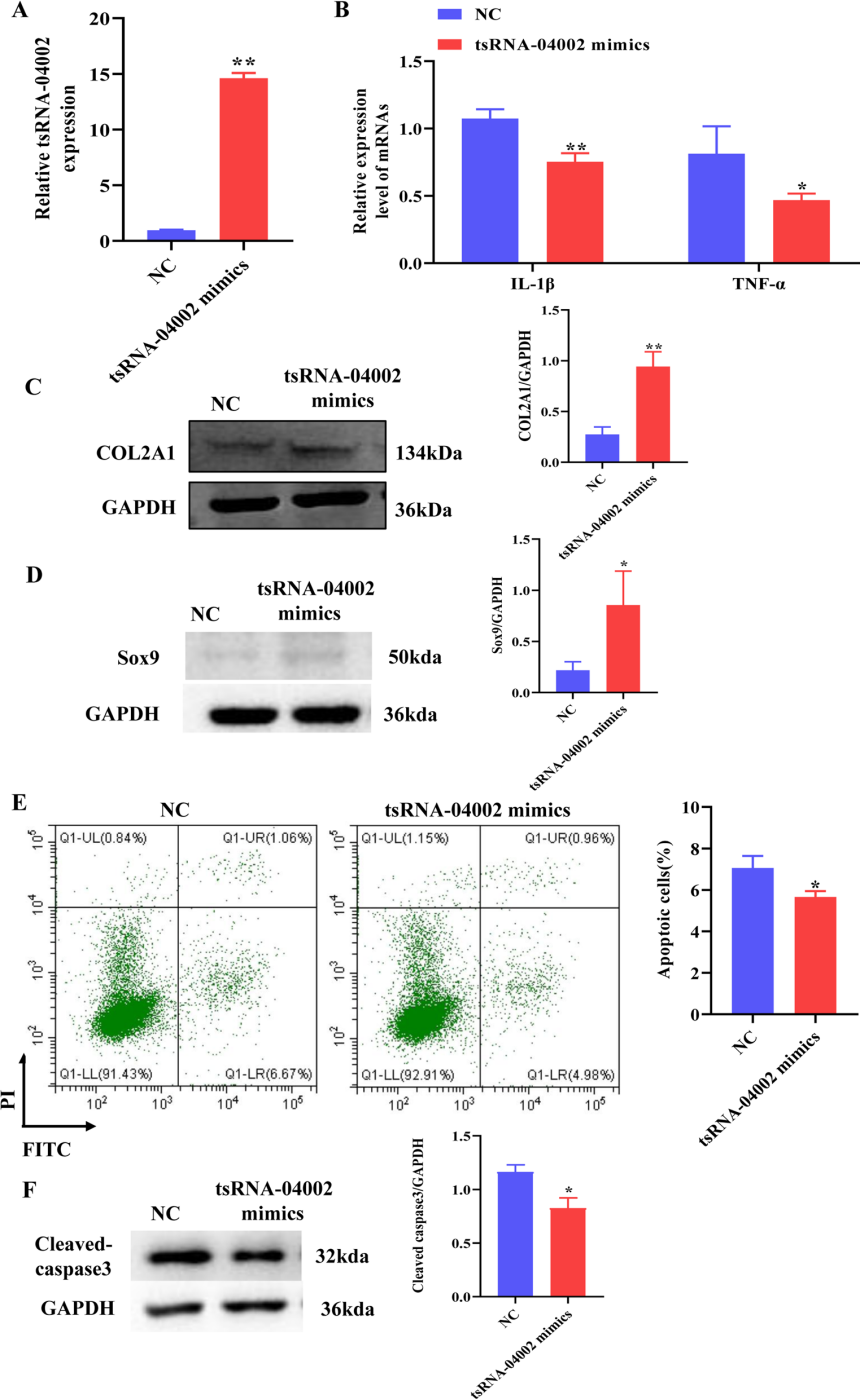
tsRNA-04002 inhibits expression of inflammatory cytokines and apoptosis of NPCs. **A** The overexpression efficiency of tsRNA-04002 was detected by qRT-PCR. **B** The levels of IL-1*β* and TNF-*α* in NPCs were evaluated using qRT-PCR. **C** Western blot analysis was applied to detect the protein expression of COL2A1 in NPCs. **D** Western blot analysis was applied to detect the protein expression of Sox-9 in NPCs. **E** Flow cytometry analysis detected the apoptosis of NPCs in tsRNA-04002 mimics NC group and tsRNA-04002 mimics group. **F** Western blot analysis was applied to detect the protein expression of Cleaved caspase3 in NPCs. *p value < 0.05, **p value < 0.01, *n* = 3

To determine the regulatory effect of tsRNA-04002 on IDD, we transfected NPCs with tsRNA-04002 mimics, certifying that tsRNA-04002 was successfully up-regulated (Fig. 3A). With the overexpression of tsRNA-04002 in NPCs, the contents of IL-1*β* and TNF-*α* in NPCs were significantly reduced by tsRNA-04002 mimics compared with the NC (Fig. 3B). In addition, the protein expression of COL2A1 was significantly up-regulated in NPCs transfected with tsRNA-04002 mimics compared with that transfected with the NC (Fig. 3C). Moreover, tsRNA-04002 mimic decreased the apoptosis of NPCs compared with corresponding negative control group (Fig. 3E). Compared with NC, the expression of Cleaved caspase3 was significantly down-regulated and the expression of Sox-9 was significantly up-regulated in NPCs transfected with tsRNA-04002 mimics (Fig. 3D, F). Together, these results indicated that tsRNA-04002 inhibited the secretion of inflammatory cytokines and inhibited apoptosis of NPCs.

### tsRNA-04002 targeted PRKCA and repressed its expression

**Fig. 4:**
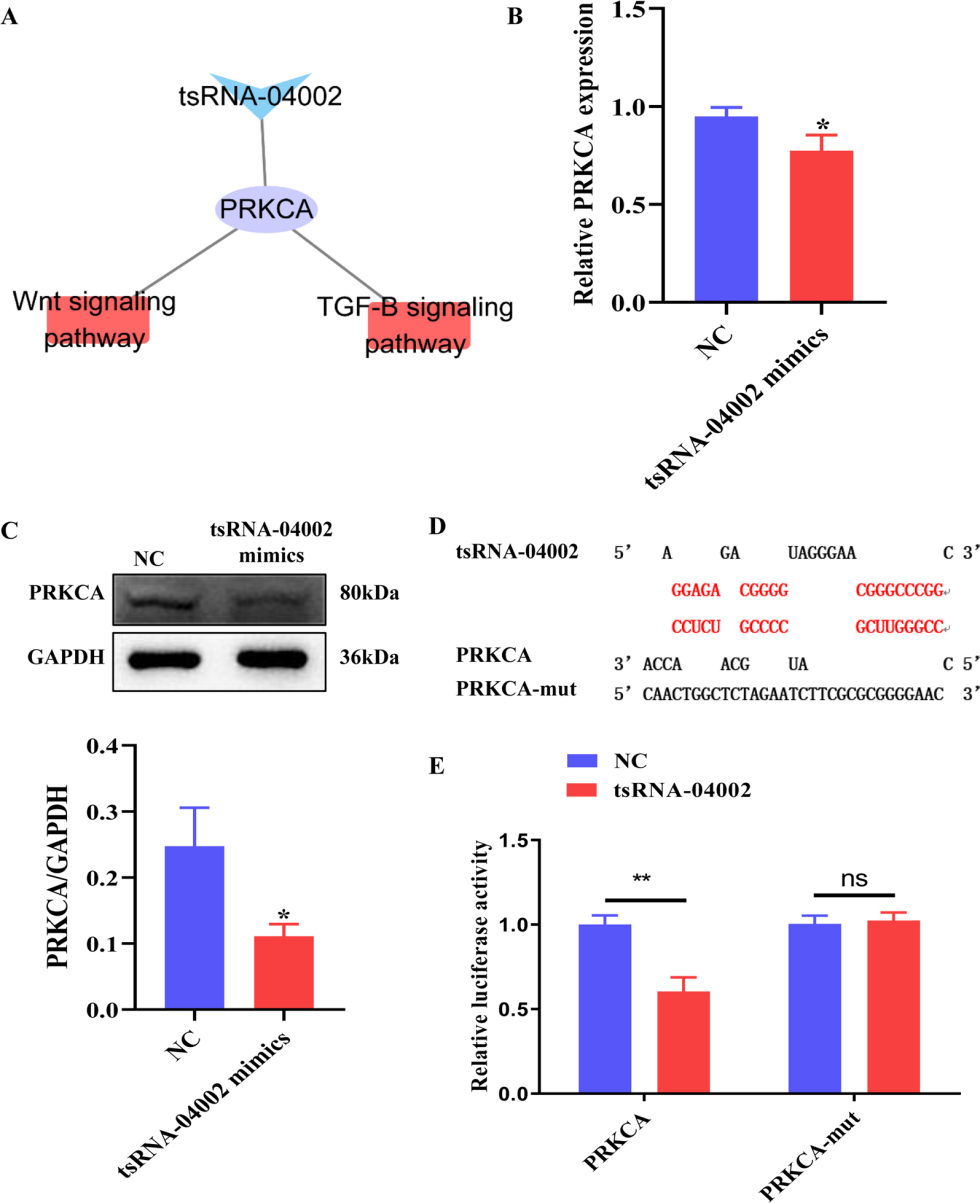
PRKCA was the target gene of tsRNA-04002. **A** Diagram of the tsRNA-04002- PRKCA-pathway network. **B** The level of PRKCA in NPCs was evaluated using qRT-PCR. **C** Western blot analysis was applied to detect the protein expression of PRKCA in NPCs. **D** Schematic representation of binding and mutation sites. **E** The relative luciferase activity was measured by dual luciferase reporter assay. *p value < 0.05, **p value < 0.01, ns: no significant significance, *n* = 3

In order to screen the target genes regulated by tsRNA-04002, we analyzed the tsRNA-04002 target genes and their signaling pathways. We found that PRKCA, a target of tsRNA-04002, was enriched in TGF-*β* signaling and WNT signaling pathway (Fig. 4A), which have been reported to be associated with IDD [25, 26]. Next, the results of RT-qPCR and western blot showed tsRNA-04002 mimics inhibited the mRNA and protein expression of PRKCA (Fig. 4B, C). To verify the direct interaction between tsRNA-04002 and PRKCA, we constructed a PRKCA fragment containing the predicted binding site (wild type and mutant) of the identified tsRNA (Fig. 4D). tsRNA-04002 mimics suppressed the luciferase activity of the WT-PRKCA compared with the NC group, while the interaction was almost abolished in the mutant group (Fig. 4E). In conclusion, these results suggested that tsRNA-04002 could bind to PRKCA and reduce its expression.

### PRKCA overexpression reverses the effect of tsRNA-04002 mimics on NPCs

**Fig. 5:**
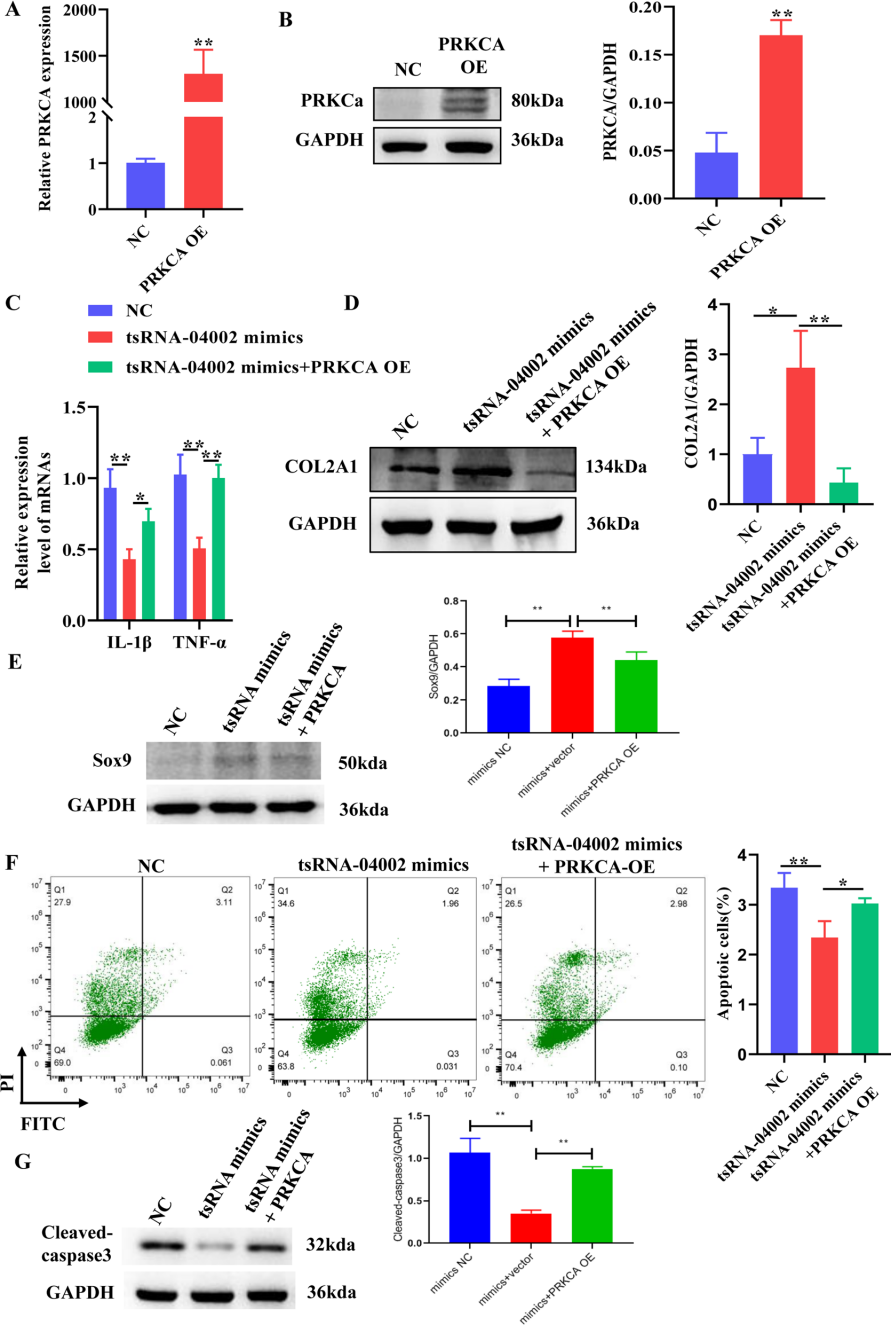
PRKCA overexpression reverses the effect of tsRNA-04002 mimics on NPCs. **A** qRT-PCR was applied for mRNA validation. **B** Protein expression of PRKCA was validated by western blot. **C** The mRNA expression of IL-1*β* and TNF-*α* was determined by qRT-PCR. **D** The protein expression of COL2A1 was determined by western blot analysis. **E** The protein expression of Sox-9 was determined by western blot analysis. **F **The apoptosis rate of NPCs was assessed by flow cytometry. **G** The protein expression of Cleaved caspase3 was determined by western blot analysis. *p value < 0.05, **p value < 0.01, *n* = 3

Based on the above findings, we further explored whether tsRNA-04002 affects IDD by regulating the PRKCA. qRT-PCR and western blot revealed that PRKCA expression level was significantly up-regulated after transfected with PRKCA overexpression plasmid (Fig. 5A, B). Here, we confirmed PRKCA overexpression neutralized the inhibitor effects of tsRNA-04002 mimics on the expression of inflammatory cytokines in NPCs (Fig. 5C). Meanwhile, compared with NC, tsRNA-04002 mimics significantly increased the protein of COL2A1 in NPCs, whereas PRKCA overexpression impaired COL2A1 expression (Fig. 5D). More importantly, the number of apoptotic cells in the tsRNA-04002 mimics group was significantly lower than that in control group, while tsRNA-04002 mimics + PRKCA OE reversed these effects (Fig. 5F). Compared with NC, tsRNA-04002 mimics significantly reduced Cleaved caspase3 expression and increased Sox-9 expression in NPCs, while these effects were reversed by tsRNA-04002 mimics + PRKCA OE (Fig. 5E, G). Overall, tsRNA-04002/PRKCA axis can exert influence on NPCs inflammation and apoptosis.

### Intradiscal injection of tsRNA-04002 alleviates IDD in a rat model

**Fig. 6:**
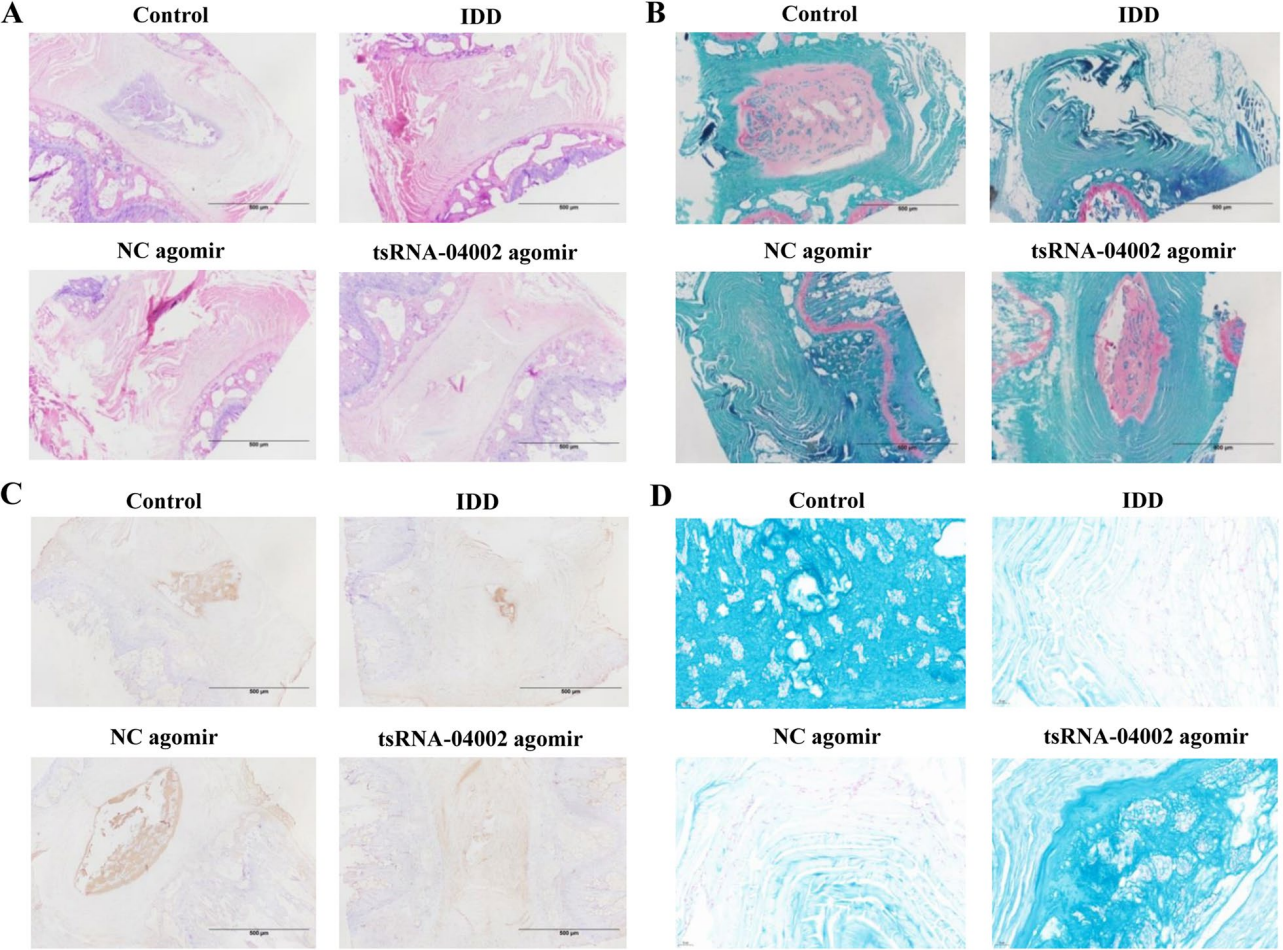
tsRNA-04002 alleviates IDD rat in vivo. **A** HE staining and **B** Safranin O-Fast green staining of the intervertebral disk. **C** The expression of PRKCA was detected by immunohistochemical staining. **D** Histological analysis of intervertebral disk tissues determined by using Alcian Blue staining. *n* = 6

To verify the function of tsRNA-04002 in IDD, we studied on its bio-functional role in IDD rat model. Histological analysis revealed that puncture-induced IDD degeneration was partially restored in IDD rat after tsRNA-04002 agomir treatment compared with IDD rat according to morphological change analysis of IDD (Fig. 6A). Safranin O-Fast Green staining demonstrated that the number of NPCs was reduced and replaced by fibrochondrocytes, and cartilage endplate collapsed in the IDD rat, while tsRNA-04002 agomir alleviated these degradations (Fig. 6B). In addition, immunohistochemical staining suggested that the expression levels of PRKCA were significantly decreased in the tsRNA-04002 agomir group compared with NC agomir group (Fig. 6C). Alcian blue staining showed that glycosaminoglycans were significantly down-regulated in the IDD model, and this downregulation was attenuated by tsRNA-04002 agomir injection (Fig. 6D). Taken together, these results demonstrated that the positive effects of elevated tsRNA-04002 on alleviates IDD by blocking PRKCA in vivo.

## Discussion

Multiple evidences indicated that certain circRNAs, lncRNAs and miRNAs could associate with the development and progression of IDD and regulate the function of NPCs by targeting specific genes [27–29]. However, the effect of tsRNAs on IDD is unclear. In this study, we first determined that tsRNA expression profile was disordered in IDD, and tsRNA-04002 as a key tsRNA involved in IDD. Mechanically, tsRNA inhibited inflammation and apoptosis of NPCs by negatively regulating PRKCA. Altogether, our findings clearly suggested that tsRNA-04002 could alleviate IDD by targeting PRKCA to inhibit apoptosis of NPCs.

Recent evidence shows that tsRNAs are dysregulated in the regulation of diseases, suggesting that tsRNAs may play an important role in the occurrence and development of diseases [30, 31]. Meanwhile, circulating nucleic acids in serum and other body fluids can be used for disease screening [32]. Therefore, we also performed small RNA sequencing on young and elderly IDD patients, and it was not unexpected to find that the tsRNA expression profile of IDD patients was disordered. Moreover, these differentially expressed tsRNAs were enriched with Wnt signaling pathway and MAPK signaling pathway. Studies have shown that the inactivation of Wnt signaling pathway can inhibit their apoptosis and inflammation of NPCs, leading to delaying IDD [33]. However, activation of MAPK/ERK signaling promoted IDD apoptosis and inflammation [34]. Therefore, dysregulated expression of tsRNA mediated Wnt signaling pathway and MAPK signaling pathway to regulate the pathogenesis of IDD.

Subsequently, we first confirmed that tsRNA-04002 was a key tsRNA in the pathogenesis of IDD, which mainly inhibited the secretion of inflammatory factors in NPCs and the apoptosis of NPCs. Previous studies indicated that multiple functional disorders in IDD development, including high expression of inflammatory cytokine, NPCs apoptosis and ECM degradation [35, 36]. Besides, studies have shown that excessive apoptosis of NPCs play an important role in the pathogenesis of IDD [37]. More importantly, our result exhibited tsRNA-04002 ameliorated the IDD process in the puncture-induced rat model. Therefore, these data suggested an important target for tsRNA-04002 to effectively alleviate IDD pathogenesis.

Noncoding RNAs generally target downstream mRNAs to affect the physiological processes of organisms. For instance, miRNA-25-3p could relieve IDD by targeting IL-1*β*/ZIP8/MTF1 [38]. CircSEMA4B as miR-431 sponge to compete with SFRP1 or GSK-3*β*, thereby inhibiting NPCs degeneration process via Wnt signaling pathway [39]. In view of this, we identified PRKCA as the binding target of tsRNA-04002, and tsRNA-04002 inhibited the apoptosis of NPCs to alleviate the development of IDD by inhibiting PRKCA expression. In addition, we found that PRKCA was enriched in TGF-*β* signaling and WNT signaling pathway. It has been reported that WNT signaling pathway activation has a significant positive correlation with IDD pathogenesis [26]. However, activation of the TGF-*β* signaling pathway inhibits disk degeneration and inflammation in the IDD rat model [25]. Cui et al. reported that inactivation of PRKCA can induce cell apoptosis [40]. Besides, PRKCA has been shown to have significant anti-inflammatory effects on some diseases, such as acute lung injury [41]. Therefore, we hypothesized that PRKCA could attenuate the inflammation of NPs by activating TGF-*β* signaling pathway and inhibiting WNT signaling pathway, thereby improving IDD. Finally, we demonstrated tsRNA-04002-treated IDD rat had reduced PRKCA expression, which implied us tsRNA-04002/PRKCA axis help future clinical IDD treatments.

In this study, we investigated the expression profile of tsRNA in NPCs from IDD patients for the first time and revealed the biological function and molecular mechanism of tsRNA-04002 in regulating NPCS degeneration, which provided a theoretical basis for further exploring the pathogenesis of IDD. In addition, this study also provides a new potential target for the treatment of IDD and provides a reference for the subsequent use of small molecule tsRNA as drug therapy, which has important scientific significance and application value.

There are some limitations of this study that need to be further explored. The mechanism of tsRNA-04002 in regulating the activity of NPCs and IDD by targeting PRKCA needs to be further elucidated. It remains unclear whether the inhibition of ECM degradation by tsRNA-04002 has an intuitive effect and what pathway tsRNA-04002 mediates to regulate IDD. Therefore, the role of tsRNA-04002 in ECM degradation and the effective signaling pathways will be explored and identified in the future.

## Conclusion

In conclusion, this study for the first time showed that tsRNA-04002 participated in the regulation of IDD. Additionally, we also demonstrated that tsRNA-04002 treatment could improve IDD rat model. Mechanistically, tsRNA-04002 significantly contributed to IDD recover by inhibiting PRKCA expression, which greatly improved our understanding of the tsRNA-04002 function.

### Supplementary Information

**Additional file 1: Table S1.** The information of primer sequence.

**Additional file 2: Fig. S1.** GO analysis of differential expressed tsRNAs. **A** Representative bubble plots of GO terms in IDDY vs Normal. **B** Representative bubble plots of GO terms in IDDO vs Normal.

## Data Availability

The data that support the findings of this study are available from the corresponding author upon reasonable request.
