# Supplementary figures and images for: tsRNA-04002 alleviates intervertebral disk degeneration by targeting PRKCA to inhibit apoptosis of nucleus pulposus cells
Source: J Orthop Surg Res. 2023 Jun 7;18:413. doi: 10.1186/s13018-023-03878-3 (PMC10249188; doi:10.1186/s13018-023-03878-3)

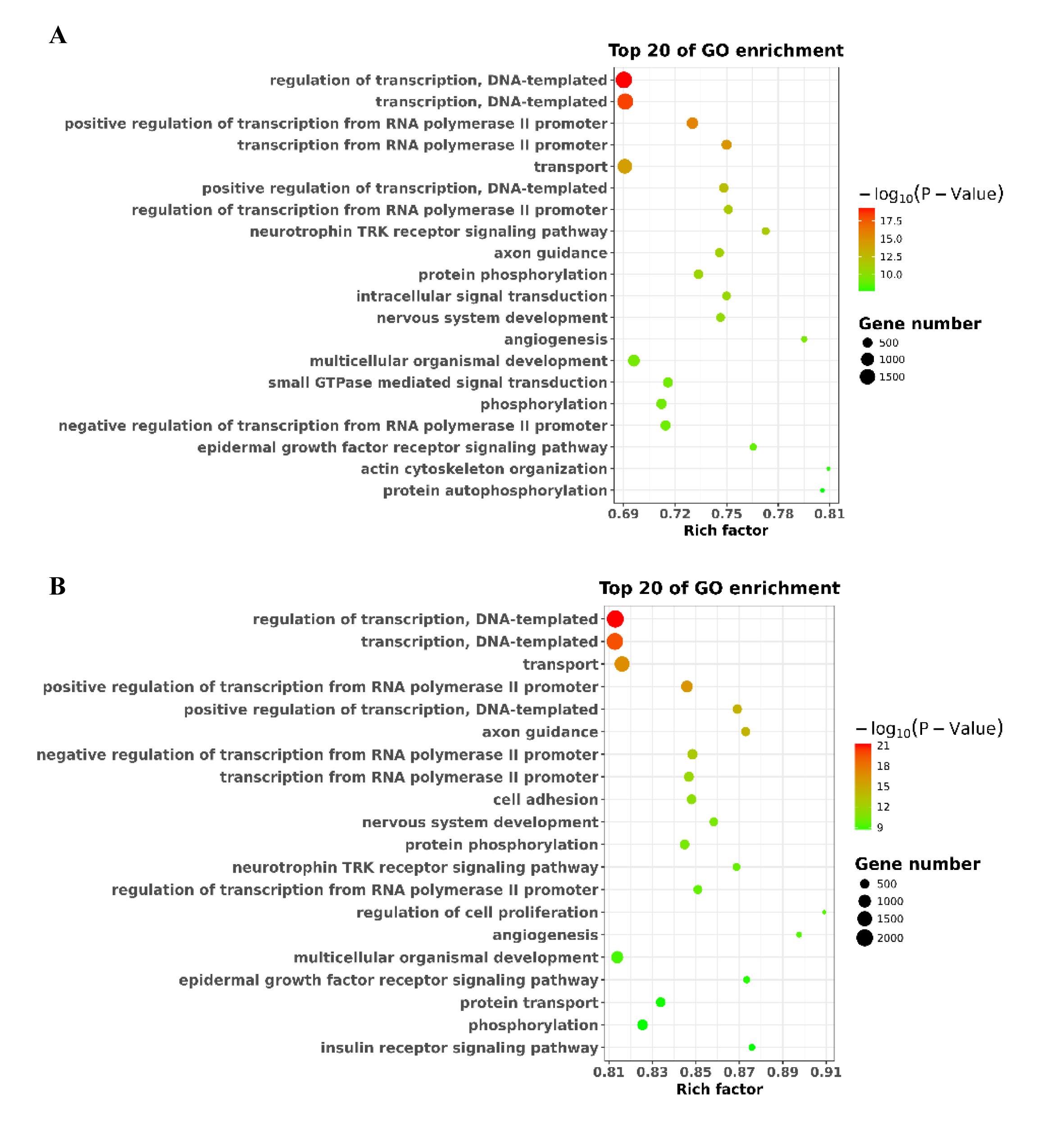

Supplement: Supplementary file 2 — Additional file 2: Fig. S1. GO analysis of differential expressed tsRNAs. A Representative bubble plots of GO terms in IDDY vs Normal. B Representative bubble plots of GO terms in IDDO vs Normal. [file 13018_2023_3878_MOESM2_ESM.tif]
